# Molecular characterization of SARS-CoV-2 nucleocapsid protein

**DOI:** 10.3389/fcimb.2024.1415885

**Published:** 2024-05-23

**Authors:** Yanping Huang, Junkai Chen, Siwei Chen, Congcong Huang, Bei Li, Jian Li, Zhixiong Jin, Qiwei Zhang, Pan Pan, Weixing Du, Long Liu, Zhixin Liu

**Affiliations:** ^1^ Department of Infectious Diseases, Renmin Hospital, School of Basic Medical Sciences, Hubei University of Medicine, Shiyan, China; ^2^ Shiyan Key Laboratory of Virology, Hubei University of Medicine, Shiyan, China; ^3^ Hubei Key Laboratory of Embryonic Stem Cell Research, Hubei University of Medicine, Shiyan, China; ^4^ Central Laboratory, The First Affiliated Hospital of Jinan University, Guangzhou, China; ^5^ Guangdong Provincial Key Laboratory of Virology, Institute of Medical Microbiology, Jinan University, Guangzhou, China

**Keywords:** COVID-19, SARS-CoV-2, nucleocapsid protein, clinical application, diagnostics

## Abstract

Corona Virus Disease 2019 (COVID-19) is a highly prevalent and potent infectious disease caused by severe acute respiratory syndrome coronavirus 2 (SARS-CoV-2). Until now, the world is still endeavoring to develop new ways to diagnose and treat COVID-19. At present, the clinical prevention and treatment of COVID-19 mainly targets the spike protein on the surface of SRAS-CoV-2. However, with the continuous emergence of SARS-CoV-2 Variants of concern (VOC), targeting the spike protein therapy shows a high degree of limitation. The Nucleocapsid Protein (N protein) of SARS-CoV-2 is highly conserved in virus evolution and is involved in the key process of viral infection and assembly. It is the most expressed viral structural protein after SARS-CoV-2 infection in humans and has high immunogenicity. Therefore, N protein as the key factor of virus infection and replication in basic research and clinical application has great potential research value. This article reviews the research progress on the structure and biological function of SARS-CoV-2 N protein, the diagnosis and drug research of targeting N protein, in order to promote researchers’ further understanding of SARS-CoV-2 N protein, and lay a theoretical foundation for the possible outbreak of new and sudden coronavirus infectious diseases in the future.

## 1 Introduction

At the end of 2019, a widespread outbreak of pneumonia cases occurred around the world. The International Committee on Taxonomy of Viruses (ICTV) formally designated the pathogen responsible for the pneumonia outbreak as Severe Acute Respiratory Syndrome Coronavirus 2 (SARS-CoV-2) on February 11, 2020. The World Health Organization (WHO) assigned the disease caused by SARS-CoV-2 infection as Coronavirus Disease 2019 (COVID-19). The emergence of COVID-19 has profoundly impacted global public health and the economy. Countries worldwide have directed their efforts towards researching the pathogenic mechanisms of SARS-CoV-2 and potential treatments (Trougakos et al., 2021; Yang and Rao, 2021; Jackson et al., 2022).

**Figure 1:**
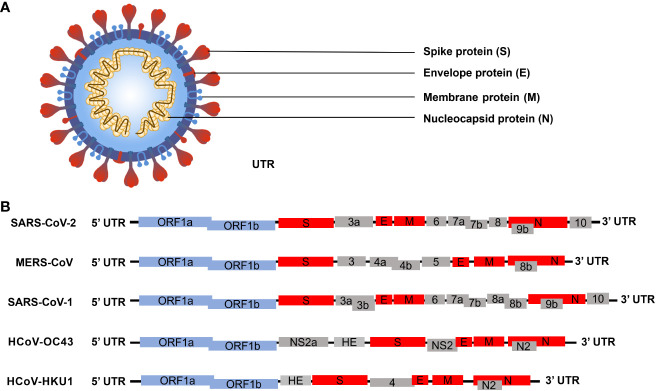
Schematic diagram illustrating the microstructural pattern and genome structure of human beta coronavirus (Chen et al., 2020b; Yang and Rao, 2021; Malone et al., 2022). **(A)** Schematic representation depicting the microstructure of beta coronavirus. The spike protein (S), membrane protein (M), and envelope protein (E) collectively assemble to form a structurally stable viral envelope. The N protein binds to the S, E, and M proteins on the viral envelope and associates with the viral RNA to form ribonucleoprotein (RNP) complexes, thereby constructing a stable viral particle. **(B)** Schematic representation illustrating the genome structures of five human beta coronaviruses. The blue boxes denote the non-structural protein-coding genes of the virus; the red boxes represent genes encoding viral structural proteins; the gray boxes indicate the genes encoding viral accessory proteins.

SARS-CoV-2 is classified within the beta coronaviruses family, and its genome exhibits similarities with other members of the beta coronaviruses (**Figure 1B**). The SARS-CoV-2 genome sequence demonstrates approximately 79% identity with the SARS-CoV genome sequence and approximately 50% identity with the MERS-CoV genome sequence (Lu et al., 2020). The SARS-CoV-2 genome, oriented from 5’ to 3’, codifies a protease and replicase coding region (ORF1ab), a Spike protein (S), a Nucleocapsid protein (N), a Membrane protein (M), and an Envelope protein (E). Additionally, it includes the 3’ untranslated region (UTRs) and a poly (A) tail (Yoshimoto, 2020). The genome comprises 14 open reading frames (ORFs) that encode 29 proteins (**Figure 1A**). The polyproteins pp1a and pp1ab are encoded within the protease and replicase coding regions. The 3C-like protease (3CLpro), encoded by Nsp5, and the papain-like protease (PLpro), encoded by Nsp3, facilitate the cleavage of the polyprotein into 16 non-structural proteins (Nsp1-Nsp16). Given that the S protein can bind to the host cell angiotensin-converting enzyme 2 (ACE2) receptor to facilitate virus entry into the host during the early stage of SARS-CoV-2 infection, coupled with its distinctive structure, it has emerged as the primary target for most diagnosis and treatment efforts at present (Liu et al., 2021b; Chen et al., 2023). SARS-CoV-2 is an RNA virus with a propensity for high mutation rate. In the context of a widespread epidemic, a substantial number of mutant strains have emerged in a relatively brief period of time (Burki, 2021; Huang and Wang, 2021; Santacroce et al., 2021). At present, the primary mutations of the novel coronavirus encompass the Alpha variant (UK), Beta variant (South Africa), Gamma variant (Brazil), and Delta variant (India). The most widespread infection was the fifth emerging South African variant (Omicron) (Cherian et al., 2021; Faria et al., 2021; Tegally et al., 2021; Volz et al., 2021).The Alpha variant consists of 17 mutations (key: N501Y) and 3 deletions. Two amino acids, H69 and V70, in the spike protein were completely deleted. Deletion of H69 and V70 increased the amount of mature spike proteins on the viral surface (Callaway, 2021; Giovanetti et al., 2021). The Beta variant contains three key mutations: N501Y, E484K, and K417N. The N501Y mutation, located in the receptor-binding domain (RBD) of the spike protein, enhances the virus’s transmissibility by promoting binding to the ACE2 receptor. The E484K mutation enables the Beta variant to evade host innate immunity more effectively. Similarly, the K417N mutation, like N501Y, affects the spike protein, facilitating its binding to the ACE2 receptor (Weisblum et al., 2020). The Gamma variant harbors numerous mutations in the spike protein, with the most concerning being N501Y, E484K, and K417T. Like the Beta variant, the Gamma variant also enhances ACE2 receptor binding and evades the human immune system through N501Y and E484K, respectively. Moreover, both the K417T and E484K mutations can augment the virus’s affinity for the ACE2 receptor, leading to enhanced binding to host cells and consequently, increased rates of infection and transmission (Jaspe et al., 2021). The Delta variant is characterized by two mutations, P681R and L452R. The P681R mutation, located at the furin cleavage site, enhances the binding of the spike protein to healthy human somatic cells. Conversely, the L452R mutation contributes to the stability of the S protein by creating a pathway that facilitates viral attachment to host cells and enhances viral replication (Kannan et al., 2021; Nanduri et al., 2021). The Omicron variant is characterized by 30 known mutations, resulting in changes in the spike protein sequence. Notably, fifteen of these mutations are located in the receptor-binding domain (RBD) of the virus. Omicron viruses exhibit increased infectivity due to mutations found in the S1-S2 cleavage sites, which are similar to those present in the Delta and Alpha variants. Among all variants of the SARS-CoV-2 virus, the Omicron variant has the highest transmission rate and severity (Ferre et al., 2022). With the ongoing emergence of SARS-CoV-2 mutants, research has indicated that mutations in the S protein exert the most significant impact on viral functional phenotypes and vaccine response, particularly on neutralizing antibodies (Yao et al., 2024). Mutations occurring in the receptor-binding domain (RBD) of the S protein induce changes in protein conformation which affect the interaction with the host ACE2 receptor (Zhang et al., 2022). Hence, greater emphasis should be placed on investigating other proteins of SARS-CoV-2, with the aim of comprehensively elucidating the virus’s structure and infection cycle, thus fostering novel insights for diagnostics and treatment. Of particular note, the N protein has high conservation and undergoes the lowest mutation rates (Chan et al., 2020; Hu et al., 2021). As a fundamental constituent of the virion, the N protein binds with viral genomic RNA and facilitates the packaging of RNA into ribonucleoprotein (RNP) complexes. This process holds significance in viral mRNA transcription and protein replication (Zeng et al., 2020). Moreover, the N protein is capable of modulating host innate immunity, rendering it a potential target for the development of early rapid diagnostic tool, as well as drug and vaccine design (Wang et al., 2021).

## 2 The structure of the N protein

**Figure 2:**
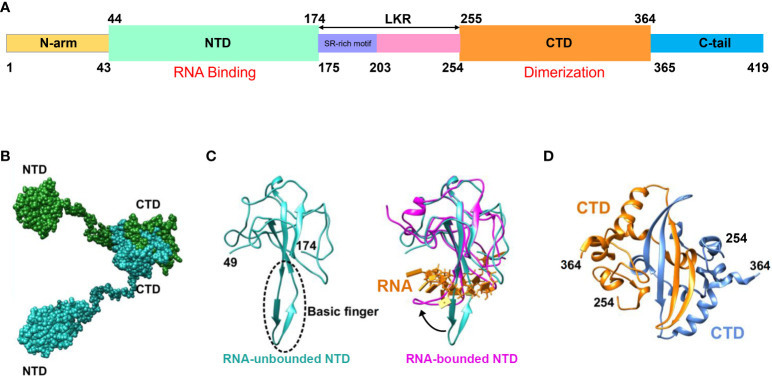
Schematic representation of SARS-CoV-2 N protein structure. **(A)** Schematic depiction of the N-protein domains. Within the NTD, basic structural motifs are present that facilitate binding to viral genomic RNA, resulting in RNP formation. CTD comprises complementary motifs that promote N-protein dimerization. **(B)** Schematic illustration of the N protein structure following dimer formation. **(C)** Schematic diagram illustrating NTD crystal structure analysis. The blue region on the left represents the resolved NTD crystal structure in the absence of RNA binding (PDB ID: 6M3M), while the area enclosed by the dashed line denotes the basic amino acid motif involved in RNA affinity. The purple segment on the right indicates the resolved NTD crystal structure post-RNA binding (PDB ID: 7ACT), whereas the yellow portion signifies the bound RNA. The black arrow denotes the three-dimensional structural alteration of the NTD basic amino acid motif following RNA binding. **(D)** Analysis of the crystal structure of CTD yielding dimer formation (PDB ID: 6WZO) (Matsuo, 2021).

The SARS-CoV-2 N protein spans 419 amino acids and encompasses three Intrinsically Disordered Regions (IDRs) along with two Conserved Structural Regions (CSRs). The IDRs consist of an N-terminal disordered structure (N-arm), a Central Ser/Arg-rich Flexible Linker Region (LKR) positioned in the middle of the protein sequence, and a C-terminal disordered structure (C-tail). The CSRs comprise the N Terminal Domain (NTD) and the C Terminal Domain (CTD) (**Figure 2A**) (Ma et al., 2021; Sarkar et al., 2022). Among these regions, the prominent β-hairpin within the NTD domain is comprised of a Basic amino acid motif (Basic finger). Its surface electrostatic potential reveals a positively charged pocket at the junction between the basic hairpin and the core structure, which facilitates binding to the viral genomic RNA and mediates viral assembly (**Figure 2C**). The CTD domains of SARS-CoV-2 have been demonstrated to form multimers (dimers, trimers, tetramers, and potentially even octamers), with the extent of aggregation dependent on the protein concentration (Peng et al., 2020; Zinzula et al., 2021). Similarly, the high-resolution crystal structure of the CTD indicates that it forms a dimer in solution owing to strand exchange induced by close contact (Peng et al., 2020; Liu et al., 2021a). Detection and analysis of the CTD domain via static light scattering and chemical cross-linking reveal that the CTD dimer remains stable in solution, and that multimomerization of this domain is instrumental in maintaining the stability of the N protein (**Figures 2B, D**) (Zeng et al., 2020).

**Figure 3:**
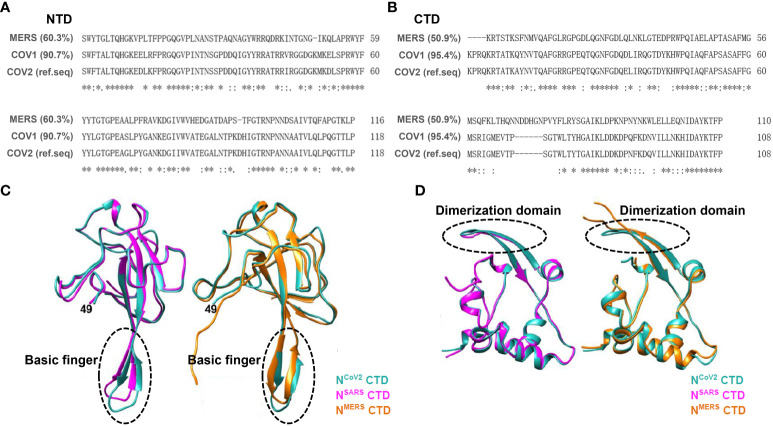
Sequence alignment and crystal structure similarity analysis were conducted on conserved domains (NTD and CTD) of the N protein across SARS-CoV-1, SARS-CoV-2, and MERS-CoV. **(A)** Aligning amino acid sequences within the NTD of three human beta coronaviruses shows significant sequence resemblance. Using NTD^COV2^ as the reference sequence, NTD^MERS^ shares 60.3% similarity, while NTD^cov1^ exhibits 90.7% similarity. **(B)** Aligning amino acid sequences within the NTD of three human beta coronaviruses shows significant sequence resemblance. Using NTD^COV2^ as the reference sequence, NTD^MERS^ shares 50.9% similarity, while NTD^cov1^ exhibits 95.4% similarity. **(C)** Comparison of the crystal structures of the N protein NTD across three beta coronaviruses. On the left, resolved structures of SARS-CoV-1 (plum red, PDB ID: 2OFZ) and SARS-CoV-2 (blue, PDB ID: 6M3M) NTD crystals are juxtaposed. On the right, comparison of resolved structures of MERS-CoV (yellow, PDB ID: 4UD1) and SARS-CoV-2 (blue, PDB ID: 6M3M) NTD crystals is presented; Gray arrows indicate basic amino acid structural motifs capable of RNA binding. The findings indicate that the NTD crystal structures across the three viral N proteins are largely identical. **(D)** Comparison of the crystal structure of CTD across three beta coronaviruses. On the left, resolved structures of SARS-CoV-1 (plum red, PDB ID: 2CJR) and SARS-CoV-2 (blue, PDB ID: 7C22) CTD crystals are compared. On the right, comparison of resolved structures of MERS-CoV (yellow, PDB ID: 6G13) and SARS-CoV-2 (blue, PDB ID: 7C22) CTD crystals is presented. The results indicate that the crystal structures of the three viral N proteins are largely consistent (Matsuo, 2021).

Based on sequence alignment analysis of SARS-CoV-2 in comparison with other coronaviruses, it is evident that SARS-CoV-2 exhibits genome sequences that are analogous to SARS-CoV-1 and MERS-CoV (**Figures 3A, B**) (Peng et al., 2020; Zhou et al., 2020). The N protein demonstrates high conservation across different coronaviruses (Chang et al., 2014; Dinesh et al., 2020; Liu et al., 2021a). Upon comparing the structures of the N protein NTD and CTD among three highly pathogenic beta coronaviruses, namely SARS-CoV-1, MERS-CoV, and SARS-CoV-2, it has been observed that their overall structures exhibit similarities (**Figures 3C, D**). However, discrepancies in the NTD structure among the N proteins of these coronaviruses mainly manifest in the β-hairpin structure associated with RNA. In comparison with N^CoV2^, the position of the β-hairpin structure of NTD in N^CoV1^ is shifted toward the N-terminus, while the β-hairpin structure of NTD in N^MERS^ is more elongated. These modifications may potentially enhance the affinity of the NTD structures of different N proteins towards RNA. Regarding the CTD structures across the three N proteins, the CTD of N^CoV2^ and N^CoV1^ exhibit similar structural characteristics, whereas the loop between the two β-layers within the N^MERS^ CTD appears longer than that of the other two N protein CTDs. Furthermore, other regions within the three N-protein CTDs display common structural features, suggesting that N-protein dimers formed via the CTD play a crucial role in RNP complex formation and RNA genome packaging (Matsuo, 2021).

## 3 Role of N protein in SARS-COV-2 replication

**Figure 4:**
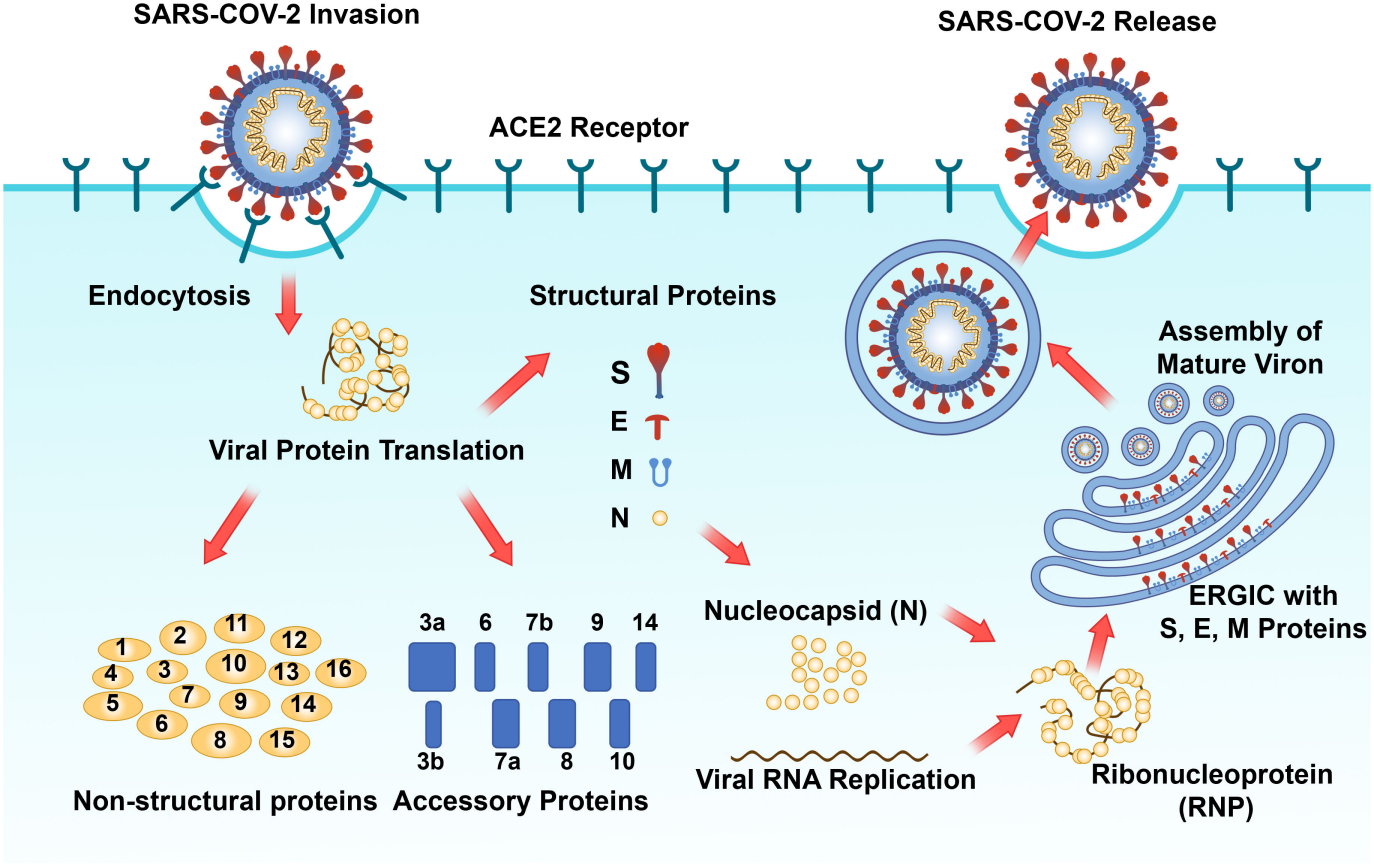
Schematic diagram of the SARS-CoV-2 replication process (Yang and Rao, 2021; Malone et al., 2022). The SARS-CoV-2 spike protein attaches to the cell ACE2 receptor, facilitating virus entry into the cytoplasm via endocytosis, where viral genomic RNA is subsequently released. Translation of viral genomic RNA within the cytosol results in the production of 16 non-structural proteins, 9 accessory proteins, and 4 structural proteins (S, E, M, and N). The N protein binds to the progeny viral genomic RNA to form RNP, which subsequently assembles into the phase separation (PS) structure and interacts with S, E, and M proteins embedded in the Endoplasmic Reticulum Golgi Intermediate Compartment (ERGIC). Mature progeny virus assembly occurs, followed by release via cell exocytosis to complete the viral replication cycle.

As one of the primary structural proteins of SARS-CoV-2, the N protein fulfills an indispensable role in the life cycle of SARS-CoV-2 (Weisblum et al., 2020; Bai et al., 2022). After the SARS-CoV-2 S protein interacts with the host cell surface receptor ACE2, the host cell molecule transmembrane protease serines 2 (TMPRSS2) facilitates the clearance of the S protein. This exposed site facilitates the fusion of the viral envelope with the host cell membrane, thus enabling viral entry into the host cell (Matsuyama et al., 2010; Walls et al., 2020). Upon viral entry, the N protein then facilitates the release of viral RNA through the capsid dissociation process (Baggen et al., 2021). Approximately two-thirds of the viral RNA genome is translated into two large polyproteins (pp1a and pp1ab) (Baggen et al., 2021). PLpro and 3CLpro catalyze the hydrolysis of these polyproteins to yield 16 mature nonstructural proteins, which subsequently assemble into viral replicase-transcriptase complexes (V'kovski et al., 2021). This complex employs (+) ssRNA as a template to synthesize (-) ssRNA and subgenomic (-) RNA via a discontinuous elongation mechanism. The previously synthesized RNA acts as a template for the generation of new plus-strand RNA, resulting in the production of subgenomic (+) mRNAs that encode structural proteins. Concurrently, the virus induces modifications to the endoplasmic reticulum (ER), transforming it into vesicular structures known as double-membrane vesicles (DMVs), which aid in the replication and translation of viral RNA (Baggen et al., 2021). Subsequently, S, E, and M proteins are transported into the ER, where the N protein binds to plus-strand RNA to form a nucleoprotein complex, thereby completing virion assembly in the Golgi apparatus (Baggen et al., 2021). Before the virion release, the hostcell´s Flynn protease cleaves five amino acid at key loci on the S protein to create infective viral particles (Menachery et al., 2020; Shang et al., 2020). Viral particles are ultimately released from infected host cells via vesicles, thereby completing their life cycle (Baggen et al., 2021). In resume, the N protein plays a pivotal role in the viral life cycle by integrating the viral genomic RNA into the ribonucleoprotein complex and facilitating the initiation of viral assembly by the M and E proteins (**Figure 4**).

## 4 The N protein’s RNA binding occurs within a phase-separated environment

Phase separation (PS) is a ubiquitous cellular process that orchestrates biomaterials into distinct compartments. In the context of virion assembly, the development of a dense protein-nucleic acid matrix is essential to sequester host cell proteins, thereby avoiding the host immune system, and to locally concentrate viral components to enhance replication efficiency (Novoa et al., 2005). It has been verified that PS is a characteristic feature of many RNA-binding proteins that possess a high proportion of intrinsic disordered regions (IDRs) (Burke et al., 2015; Sanders et al., 2020). The SARS-CoV-2 N protein possesses a positive charge, enabling its interaction with negatively charged viral genomic RNA, thereby facilitating the assembly of RNA into a more structured configuration. Upon RNA binding, the SARS-CoV-2 N protein readily undergoes PS, resulting in the formation of liquid droplets whose phase behavior is substantially affected by pH, salt concentration, and RNA concentration (Cascarina and Ross, 2020; Iserman et al., 2020; Perdikari et al., 2020; Savastano et al., 2020; Jack et al., 2021). The N protein associates with the viral genomic RNA to form RNP complexes, initiating viral assembly. Following viral infection, PS facilitates the formation of stress granules (SG) and processing bodies (PBs) by disrupt viral mRNA translation and promote RNA decay. The development of SG significantly suppresses viral protein translation, thereby hindering viral gene expression (Mccormick and Khaperskyy, 2017). Ras-GTPase-activating protein binding protein 1 (G3BP1) and Ras-GTPase-activating protein binding protein 2 (G3BP2), which are linked to mRNA, serve as vital constituents of SG. G3BP1 exerts inhibitory effects on viral replication by actively modulating RIG-I and cGAS-mediated cellular antiviral responses (Liu et al., 2019; Yang et al., 2019). The N protein can engage with G3BP to suppress type I interferon (IFN) signaling and G3BP-mediated SG formation, while promoting viral infection through the neutralization of G3BP1-mediated antiviral innate immunity (Liu et al., 2022). The PS of the N protein facilitates the virus-induced activation of nuclear factor kappa-B (NF-κB) signaling. Upon binding to viral RNA, the N protein undergoes PS to recruit transforming growth factor-β-activated kinase 1 (TAK1) and inhibitor of kappa B kinase (IKK) complexes, which are pivotal kinases involved in NF-κB signaling, resulting in the augmentation of NF-κB activation. Consequently, this elicits an inflammatory response and the secretion of various cytokines (Wu et al., 2021).

## 5 Host immunity modulation of by N protein

The innate immune system represents the host’s primary defense against microbial infections, enhancing host immunity through the recognition and elimination of infected cells, as well as the regulation of the immune response in viral infections (Shibabaw et al., 2020). RNA interference (RNAi) represents a conservative antiviral immune defense mechanism present within host cells, capable of resulting in the degradation of viral genomes and the inhibition of viral replication. RNAi demonstrates significant antiviral efficacy across fungi, plants, and animals (Zhang et al., 2020). Viruses typically encode RNA silencing suppressors (RSSs) to subdue host RNAi responses and evade host immune defenses. Previously, it has demonstrated that the N protein of SARS-CoV possesses notable RSS activity in mammalian cells (Cui et al., 2015). SARS-CoV-2 exhibits a considerable amino acid identity (94%) with the N protein of SARS-CoV, with the critical residues Lys 258 and Lys 262 for viral suppressor of RNA silencing (VSR) activity remaining conserved in the SARS-CoV-2 N protein. Other reports have indicated that the SARS-CoV-2 N protein can also act as a VSR in both the initiation and execution phases of host cell RNAi, emerging as a pivotal immune evasion factor of SARS-CoV-2 (Mu et al., 2020b).

Type I interferon (IFN) serves as the primary defense against viruses and is instrumental in instigating the host’s antiviral response (Kato et al., 2006; Catanzaro et al., 2020). There are two principal pathways that activate type I IFN: the RIG-I-like receptor (RLR) pathway and the Toll-like receptor (TLR) pathway (Chan and Gack, 2016). Double-stranded viral RNA and short dsRNA containing 5’ -triphosphate are detectable by RIG-I-like receptors (RLR), including retinoic acid-inducible gene 1 (RIG-1) and melanoma differentiation-associated protein 5 (MDA5) (Rehwinkel and Gack, 2020). RIG-I identifies viral RNA and subsequently undergoes dephosphorylation, thus initiating RIG-I polyubiquitination via the ubiquitin E3 ligase triple motif protein 25 (TRIM25) (Okamoto et al., 2017). Dimerization of mitochondrial antiviral signal transduction proteins (MAVS, also known as IFN-β promoter stimulator I) is triggered by the interaction of deubiquitinated RIG-1 with the caspase recruitment domain. MAVS dimerization interacts with the TNF receptor-associated factor (TRAF) domain and TRAF interaction motif, respectively, facilitating the binding of TRAF3. TRAF3 then recruits adaptor proteins NAP1 (Nucleosome Assembly Protein 1), TANK (TRAF Family Member Associated NFKB Activator), and SINTBAD (similar to NAP1 TBK1 adaptor). TANK is capable of bridging upstream RLR signaling to TANK-binding kinase 1 (TBK1), leading to the phosphorylation of interferon regulatory factor 3 (IRF-3) by TBK1 (Verhelst et al., 2013). Phosphorylation and dimerization of IRF-3 triggers nuclear translocation of IRF-3, after which IRF-3 binds to interferon-stimulated response elements (ISRE) to enhance type I interferon gene expression (Schneider et al., 2014). It has been demonstrated that the SARS-CoV-2 N protein interacts with the DExD/H domain of RIG-I to inhibit the phosphorylation and nuclear translocation of interferon regulatory factor 3 (IRF3), thereby hindering the activation of IRF3 and ultimately resulting in the failure of RIG-mediated IFN-β production (Chen et al., 2020a). PS induced by the N protein can impede the polyubiquitination and aggregation of MAVS, thus restraining the antiviral immune response (Wang et al., 2021). After interacting with TRIM25, the N protein inhibits TRIM25-mediated RIG-I ubiquitination and activation, which impacts the interferon response (Hu et al., 2017). The N protein can additionally inhibit the phosphorylation and nuclear translocation of transcription factors signal transducer and activator of transcription 1 (STAT1) and transducer and activator of transcription 2 (STAT2), resulting in the down-regulation of Interferon-stimulated gene factor 3 (ISGF3), which antagonizes IFN1 production and ultimately hinders the host innate immune response, thereby impairing the immune system’s ability to combat viruses (Mu et al., 2020a).

**Figure 5:**
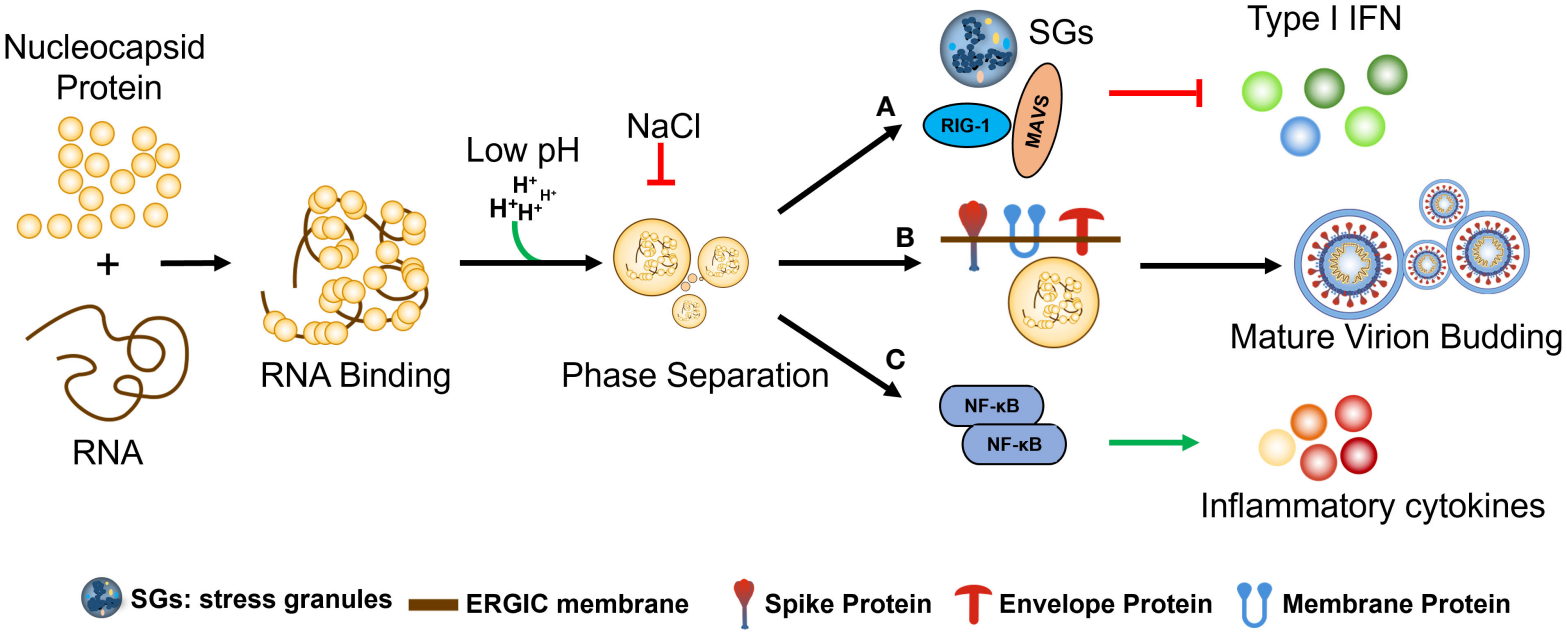
The regulation and functional significance of SARS-CoV-2 N protein phase separation. The phase separation of the N protein is initiated by its binding with RNA molecules of various lengths and is influenced by electrostatic forces such as pH and NaCl concentration. **(A)** The phase separation of the N protein has several effects, including the formation of stress granules (SGs) and interaction with key factors involved in the host cell’s antiviral response, such as MAVS and RIG-I, ultimately leading to the inhibition of type I interferon (IFN) production. **(B)** During the assembly and budding of mature virions, the N protein interacts with viral genomic RNA (gRNA) to form condensates, which are then anchored at the ERGIC membrane through co-phase separation (co-PS) with the SARS-CoV-2 M protein. **(C)** The N protein phase separation also regulates host-cell innate immune pathways by interacting with NF-κB activators, resulting in the upregulation of cytokine production. This, in turn, elicits an inflammatory response and the secretion of various cytokines, further contributing to the host immune response against SARS-CoV-2 infection.

N protein phase separation occurs by binding with RNA of various lengths and influencing by electrostatic forces (pH and Nacl etc.). (a) N protein phase separation effects stress granules (SGs) and anti-RNA virus key factor (MAVS AND RIG I) TO inhibit host-cell type I IFN. b) anchoring of N+gRNA condensates at the ERGIC membrane by co-PS with the SARS-CoV-2 M protein during Assembly and budding Mature virion; c) regulation of host-cell innate immune pathways, including upregulation of cytokine production via co-PS with NF-κB activators elicits an inflammatory response and the secretion of various cytokines (**Figure 5**).

## 6 Diagnostics and drug development targeting N protein

Given the pivotal role of the N protein in SARS-CoV-2 replication and infection, several studies have illustrated that it can serve as an effective target for diagnosing and treating SARS-CoV-2.

RT-qPCR technology is highly sensitive to the viral genome and is widely regarded as the “gold standard” for COVID-19 diagnosis (Wang et al., 2020). Given the low mutation rate observed in the N protein during SARS-CoV-2 mutations, nucleic acid detection of SARS-CoV-2 can directly target the N gene. Nucleic acid detection kits specifically designed for the N gene are also currently in development (Farfour et al., 2021). In response to the ongoing evolution of SARS-CoV-2 variants, Vega-Magana et al. devised three specific primers and probes targeting mutation sites 69–70Del and K417N within the N gene. These tools enable the specific screening and detection of SARS-CoV-2 variants using RT-qPCR methodology (Vega-Magana et al., 2021). Reverse transcription loop-mediated isothermal amplification (RT-LAMP) and reverse transcription recombinase polymerase amplification (RT-RPA) are highly sensitive and specific techniques for detecting SARS-CoV-2, serving as viable alternatives to RT-qPCR (Kashir and Yaqinuddin, 2020; Alhamid et al., 2022). In their study, Almeida et al. utilized RT-LAMP targeting the N and E genes to detect a broad spectrum of Omicron variants (Almeida et al., 2022). Jose L et al. devised two RT-RPA assays tailored for detecting deletion-insertion mutations unique to the Omicron BA.1 variant within the partial N and S genes of SARS-CoV-2 (Malaga et al., 2023). The N protein is abundantly expressed in the host following viral infection. Due to its significant conservation, the N protein serves as one of the most dependable early markers and is also a sensitive and specific indicator for serological detection (Grifoni et al., 2020; Okba et al., 2020). The detection of targeted N antigen and specific antibodies can help mitigate the occurrence of false-negative results in RT-qPCR assays. Currently, the N protein is extensively employed for rapid antigen detection of SARS-CoV-2. Diao et al. have devised a rapid method utilizing fluorescence immunochromatography (FIC) for SARS-CoV-2 N antigen detection. Comparative analysis with RT-PCR demonstrates that FIC for NP antigen detection exhibits superior specificity and sensitivity in the early diagnosis of SARS-CoV-2 infection (Grifoni et al., 2020). As a vital method in serological detection, enzyme-linked immunosorbent assay (ELISA) enables the diagnosis and quantification of infection intensity by detecting antibodies generated during viral infection. Employing ELISA, Ogata et al. discovered that 64.1% of COVID-19-positive patients exhibited SARS-CoV-2 N antigen presence in their plasma (Ogata et al., 2020). Cai et al. devised an ultra-sensitive, rapid, double-digital enzyme-linked immunosorbent assay (dELISA) based on a single-molecule array targeting the spike protein (S-RBD) and N protein to enhance the precision of COVID-19 diagnosis (Cai et al., 2021). Tan et al. established a microfluidic chemiluminescence ELISA platform capable of detecting S and N proteins in 10-fold diluted serum within 40 minutes (Tan et al., 2020).

Based on the structure, function, and life cycle of the N protein, it also represents a potential drug target for the treatment of COVID-19 (Matsuo, 2021). The RNA-binding activity of the N protein is crucial for viral RNP formation and genome replication. Therefore, the development of drugs capable of blocking the RNA-binding activity of NTD and CTD represents a promising antiviral strategy (Peng et al., 2020). Recent studies have shown that a novel compound K31 can bind to SARS-CoV-2 N protein in a non-competitive manner, thereby inhibiting its binding to the 5’ end of viral genomic RNA and reducing viral replication (Royster et al., 2023). Through drug screening, some research teams have discovered that ceftriaxone sodium, a third-generation antibiotic, is a small molecule compound capable of binding to both NTD and CTD. Because ceftriaxone inhibits RNP formation by blocking RNA binding to NTD, it could potentially impede SARS-CoV-2 replication (Luan et al., 2022). There are also studies investigating the stability of NTD-drug complexes using molecular docking and molecular dynamics simulations to identify potential antiviral drugs (Tatar et al., 2021). The new strategy of repurposing old drugs can also be used to treat SARS-CoV-2 in a very short time and at low cost. Thirty-four drugs approved or under development, such as rapamycin, salacatinib, and carolimstat, have shown varying degrees of antiviral effects (Matsuo, 2021). In addition, some screened inhibitors could disrupt N protein oligomerization to prevent RNP formation or induce abnormal aggregation. 5-benzyloxyrrhizine was found to enhance the stability of the N-NTD dimer through hydrophobic interaction, leading to abnormal N protein oligomerization (Lin et al., 2020). Previous studies have shown that folic acid can play an antiviral role by antagonizing the VSR activity of N protein (Chen et al., 2022). In our previous study, our group also screened a ssDNA aptamer targeting N protein, N-APT17, which can also inhibit the PS of N protein by binding to N protein and reduce viral replication and infection (Huang et al., 2024). In summary, the N protein can be an effective target for SARS-CoV-2 diagnosis and drug development, and drug research targeting the N protein is crucial.

## 7 Progress in N protein vaccine development

Since the outbreak of the coronavirus disease 2019 (COVID-19), SARS-CoV-2 has undergone numerous mutations in its genome, with mutations in the S protein being particularly prominent (Yao et al., 2024). A study found that among 104 representative amino acid mutations in the S protein, 21 mutations were found to enhance infectivity, while 19 amino acid mutations reduced infectivity, and 43 amino acid mutations increased the potential for immune escape (Zhang et al., 2022). Among them, 14 mutations enhanced both infectivity and immune escape, and 18 mutations enhanced immune escape at the cost of reducing transmission. Additionally, vaccines targeting the S protein may promote pericyte (PC) dysfunction, leading to microvascular damage. PCs are parietal cells that maintain and repair the entire myocardial microvascular system. S protein induces dysfunction of PCs through interaction with the CD147 receptor on PCs, which promotes the release of pro-apoptotic factors and induces the death of vascular endothelial cells. At the same time, S protein induces PCs to release proinflammatory cytokines, damaging adjacent cardiomyocytes and activating the intima, promoting blood coagulation, increasing vascular permeability, and eventually inducing related cardiovascular complications (Avolio et al., 2021). Therefore, considering the aforementioned two major drawbacks of S protein-targeted vaccines, the N protein has garnered significant attention as a vaccine development target. Compared with the S protein, the key domains of the N protein remain highly conserved across different variants. Additionally, a large number of antibodies and IFN could also be produced in the host after N protein antigen inoculation, inducing a robust immune response in host cells (Sun et al., 2021; Feng et al., 2022). Vaccines containing the full-length N protein have been demonstrated to be effective against SARS-CoV-2 variants (Wang et al., 2022). Additionally, these vaccines with full-length N protein have exhibited high tolerance in clinical trials (Chiuppesi et al., 2022b), and some of them have been shown to produce neutralizing antibodies that strongly bind to the N protein (Jia et al., 2021; Wang et al., 2022). Messenger RNA (mRNA) vaccines that express a more conserved viral N protein (mRNA-N) can reduce viral load when administered alone, while combining mRNA-N with an S-expressing mRNA vaccine (mRNA-S +N) can effectively target Delta and Omicron variants (Hajnik et al., 2022). However, as SARS-CoV-2 variants continue to emerge, new strains are gradually evading neutralizing antibodies. T cell responses are increasingly recognized as playing a pivotal role in controlling infection and reducing virus transmission (Tarke et al., 2021; Liu et al., 2021a). The N protein has been identified to harbor crucial T-cell epitopes that are integral for immunity against SARS-CoV-2 (Kiyotani et al., 2020; Lee et al., 2021). N proteins activate T cell responses via Fc receptors and participate in phagocytosis during infection, a process pivotal to the innate immune system (Lopez-Munoz et al., 2022; Maghsood et al., 2023). Hence, N protein T-cell epitopes hold promise for developing vaccines with broad applicability against SARS-CoV-2. Appelberg et al. engineered a SARS-CoV-2 DNA vaccine incorporating receptor-binding domain loops from huCoV-19/WH01, Alpha, and Beta variants, while targeting M and N proteins. This vaccine elicited spike antibodies that cross-reacted to neutralize huCoV-19/WH01, Beta, Delta, and Omicron viruses *in vitro*, while also stimulating N protein-specific T cells. The protective efficacy of solely priming cross-reactive nucleoprotein-specific T cells reached 60% (Appelberg et al., 2022). A chimeric protein vaccine (SpiN), containing the receptor binding domain (RBD) of the spike (S) protein along with the N antigen, has been found to protect against Delta and Omicron variant infections by eliciting a robust IFN-γ response with T cells and high levels of inactivated virus antibodies, with no neutralizing antibodies detected *in vivo* (Castro et al., 2022). In conclusion, vaccines targeting the N protein show great potential in preventing infections caused by wild-type SARS-CoV-2 and its variants, yet further exploration is needed (**Table 1**).

**Table 1:** The research progress of SARS-CoV-2 nucleocapsid protein vaccine.

Vaccine name	Vaccine type	N-protein usage	Animal or human experiment	References
Ad5-N	Viral Vectored Vaccine	Full length N-protein from SARS-CoV-2 ancestral virus, with co-expression of S protein.	Mouse	(Dangi et al., 2021)
MVA/SdFCS-N	Viral Vectored Vaccine	Full length N-protein from SARS-CoV-2 ancestral virus, with co-expression of S protein.	Rhesus macaque, Mouse	(Routhu et al., 2022)
rACAM2000SN	Viral Vectored Vaccine	Full length N-protein from SARS-CoV-2 ancestral virus, with co-expression of S protein.	Hamster	(Deschambault et al., 2022)
rLVS*Δ capB*/SCoV2 MN	LVSΔ*capB* Vectored Vaccine	Full length N-protein from SARS-CoV-2 ancestral virus, with co-expression of M protein.	Hamsters and mice	(Jia et al., 2021)
GX-19N	Recombinant DNA vaccine	Full length N-protein from SARS-CoV-2 ancestral virus, with co-expression of S protein.	healthy adult	(Ahn et al., 2022)
UB-612	Multitope subunit vaccine	Rationally designed promiscuous peptides representing sarbecovirus conserved CD4 ad CD8 T cell epitopes on N, M, and S2 proteins, and S1-RBD-sFc protein.^++^	healthy adult	(Wang et al., 2022)
RBD-P2/N	Recombinant Protein Vaccine	Full length N-protein from SARS-CoV-2 ancestral virus, with the receptor binding domain (RBD) of the spike protein fused with the tetanus toxoid epitope P2	Mouse, Rat, NHP	(Hong et al., 2021)
Tri: ChAd	Viral Vectored Vaccine	Full length N-protein from SARS-CoV-2 ancestral virus, in fusion witha highly conserved region of RNA-dependent RNA polymerase and with co-expression of S1 domain of S protein.	Mouse	(Afkhami et al., 2022)
COH04S1	Viral Vectored Vaccine	Full length N-protein from SARS-CoV-2 ancestral virus, with co-expression of S protein.	Hamster, NHP	(Chiuppesi et al., 2022a)
mRNA-S + N	RNA vaccine	A mRNA vaccine expressing fulll length N-protein from SARS-CoV-2 ancestral virus, alone or in combination withthe clinically proven S-expressing mRNA vaccine.	Mouse, Hamster	(Hajnik et al., 2022)
SpiN	Recombinant Protein Vaccine	Full length N-protein from SARS-CoV-2 ancestral virus, in fusion with RBD of S protein.	Mouse, Hamster	(Castro et al., 2022)

## 8 Prospects and discussion

SARS-CoV-2 continues to circulate and has become a common pathogen, and the fact that SARS-CoV-2 is still mutating and evolving new adaptation mechanisms makes combating this disease an ongoing challenge (Cong et al., 2020). Therefore, the life cycle of SARS-CoV-2, including viral replication and infection, still needs to be explored. Among them, the N protein stands out as highly conserved in SARS-CoV-2 development and plays a pivotal role in viral infection, making it a prime research target against SARS-CoV-2. Currently, numerous studies are underway to screen and develop targeted drugs for the N protein, aiming to disrupt its interactions with viral RNA, thereby impeding virus packaging and replication. Additionally, these studies explore the impact of the N protein on host cell innate immunity.

Additionally, considering that the key domains of the N protein remain highly conserved across different variants, vaccination with N protein antigens can stimulate the host to produce a plethora of antibodies and interferons (IFNs), leading to a robust immune response in host cells. Consequently, research on SARS-CoV-2 vaccines targeting the N protein has yielded remarkable results. This article reviews the current research progress of the N protein of SARS-CoV-2, hoping to deepen people’s understanding of the structure and function of the N protein of SARS-CoV-2 and the role it plays in the viral life cycle. At the same time, the potential of the N protein in the development of diagnostics, drugs, and vaccines is discussed.

Previously, due to the key role of the S protein in the process of viral invasion, several approved COVID-19 vaccines have been selected as the main immunogens from the S protein or its receptor binding domain (RBD) (Chen et al., 2021; Wang et al., 2021). However, with the continuous emergence of SARS-CoV-2 variants, the key receptor domains in the S protein are also constantly mutated, which brings great challenges to the existing vaccines and vaccine research and development. In view of this concern, international attention should be paid to the more conservative N protein. The characteristics of high immunogenicity and abundant expression during infection make N protein indispensable for SARS-CoV-2 replication and infection, and N protein plays a very important role in the pathogenic process of the virus. Therefore, N protein can also be used as an important target for SARS-CoV-2 diagnosis and drug development. Hence, the N protein emerges as a crucial target for the diagnosis, drug development, and vaccine formulation against SARS-CoV-2 and its variants. Moreover, it holds potential for immune defense against other coronaviruses closely related to SARS-CoV-2, such as MERS and SARS.

In summary, the pathogenesis of SARS-CoV-2 infection and the molecular mechanisms underlying virus-host interactions need to be explored. Tackling this epidemic is a long-term effort that requires the joint efforts of all sectors.

## Author contributions

YH: Writing – original draft, Data curation. JC: Writing – original draft. SC: Writing – original draft. CH: Writing – original draft. BL: Writing – original draft. JL: Writing – original draft. ZJ: Writing – original draft. QZ: Writing – original draft. WD: Writing – original draft. LL: Writing – original draft. ZL: Writing – review & editing, Visualization, Supervision, Funding acquisition, Data curation. PP: Writing – original draft, Data curation.
